# Red Meat Consumption, Iron Status, and Cardiometabolic Risk in Qatari Adults: A Cross-Sectional Gender-Stratified Analysis from the QPHI-QBB Data in Qatar

**DOI:** 10.3390/foods14122134

**Published:** 2025-06-18

**Authors:** Hanaa Mousa, Nadin M. Abdel Razeq, Yasmen Khial, Reema Tayyem

**Affiliations:** 1 Vice President for Health and Medical Sciences Office, QU Health, Qatar University, Doha 2713, Qatar; hanaa.mousa@qu.edu.qa; 2 College of Nursing, QU Health, Qatar University, Doha 2713, Qatar; nadin@qu.edu.qa; 3 Nutrition Sciences Department, College of Health Sciences, QU Health, Qatar University, Doha 2713, Qatar; yk1703045@student.qu.edu.qa

**Keywords:** red meat, iron profile, metabolic parameters, Qatar Biobank

## Abstract

Background: Red meat, a significant source of heme iron, may influence iron status and metabolic health, particularly in Qatar, where consumption is high. Understanding these associations is essential for addressing iron deficiency and cardiovascular risk in this population. Methods: We conducted a cross-sectional study using data from 13,778 Qatari adults enrolled in the Qatar Biobank (men: *n* = 5770; women: *n* = 8008). Red meat intake was assessed via the Food Frequency Questionnaire and categorized as low (≤1/month), moderate (2–4/month), and high (≥5/month) intake. Hematological and metabolic biomarkers were analyzed. Two-sample *t*-tests compared biomarker levels by gender. Multiple linear regression examined associations between red meat intake and iron profile indicators, adjusting for age, gender, supplement use, diabetes, hypercholesterolemia, and hypertension. Results: High red meat consumption was associated with increased ferritin (Coef = 134.685, *p* < 0.001) and hemoglobin (Coef = 0.918, *p* = 0.017). Males showed higher hemoglobin (14.8–14.9 vs. 12.4–12.5 g/dL, *p* < 0.0001) and total cholesterol (5.17 ± 1.10 vs. 5.02 ± 1.01 mmol/L, *p* = 0.0125). TIBC showed no significant gender differences across categories (*p* > 0.15) but varied significantly within each gender across red meat consumption categories (males: *p* < 0.0000; females: *p* < 0.0000). Conclusions: Higher red meat intake is associated with improved iron status, particularly ferritin levels, and gender-specific effects on hemoglobin and cholesterol levels. Moderate red meat intake may support iron health while maintaining a favorable lipid profile.

## 1. Introduction

Meat is a crucial component of human diets, serving as a rich source of protein, essential amino acids, minerals, vitamins, and other micronutrients [1]. Among meat types, red meats such as beef and lamb are particularly notable for being the richest dietary sources of heme iron, the most bioavailable form of iron [2]. Consuming red meat can significantly enhance iron status, making it especially beneficial for individuals at risk of iron deficiency anemia (IDA) [1]. Iron plays a vital role in blood formation, particularly in the synthesis of hemoglobin. Inadequate iron intake may lead to anemia [3], which is defined by the World Health Organization (WHO) as a condition characterized by a low number of red blood cells or low hemoglobin levels in the blood [4].

One of the most consistent patterns in global dietary behavior is the gender-based difference in meat consumption, with men typically consuming significantly more meat, especially red and processed meats, than women [5]. This higher intake contributes to improved iron status in males, given the high bioavailability of heme iron in red meat. By contrast, females tend to have greater variability in iron levels due to factors such as menstrual blood loss during reproductive years and hormonal changes associated with menopause [5]. As a result, women are more vulnerable to IDA, while young men often exhibit higher serum ferritin concentrations, although this sex-based difference narrows with age. Additionally, the combination of lower meat consumption and increased iron requirements places women, particularly premenopausal women, at greater risk of IDA [5].

Iron status also varies by sex and across different life stages. In females, iron levels are influenced by blood loss during menstruation and undergo further changes with the onset of menopause [6]. As a result, females experience greater fluctuations in iron levels compared to males. Typically, young males exhibit higher serum ferritin concentrations than age-matched females; however, this sex-based difference tends to narrow with advancing age, particularly after menopause [6]. Iron in the diet comes in two forms: **heme iron**, found in animal products, which is highly bioavailable, and **non-heme iron**, found in plant-based foods, which is less bioavailable [2]. Consuming heme iron can enhance the absorption of non-heme iron, highlighting the greater risk of IDA among vegetarians and vegans [7]. Although it has nutritional benefits, red meat has been associated with adverse health outcomes. Several studies have linked high consumption of red and processed meats with increased risk of metabolic syndrome [8], **cardiovascular diseases** [9], and certain types of **cancers** [10]. A metabolomics study identified 46 out of 225 metabolites associated with red meat intake [9]. These included positive associations with various lipoproteins, lipid components (cholesterol, phospholipids, and apolipoproteins), and other compounds such as fatty acids, choline, and phosphoglycerides. Notably, 29 of these markers were also associated with increased cardiovascular disease (CVD) risk, with both sets of associations generally aligned in direction [9]. These associations may be linked to the negative effects of animal protein [11]. The high iron content in red meat, particularly heme iron [12], and its saturated fat content, specifically long-chain saturated fatty acids, may synergistically contribute to cardiovascular risk [13,14].

The most recent national dietary data from the 2012–2013 Qatar National Household Income and Expenditure Survey (HIES) indicates distinct patterns in meat consumption: white meat (poultry, fish, and seafood) accounted for 57% of total meat intake, while red meat products comprised the remaining 43%. This distribution reflects Qatar’s unique dietary landscape, where lamb (constituting 30% of red meat consumption) remains culturally prominent despite higher overall white meat intake [15]. Given Qatar’s unique cultural and dietary context, understanding the relationship between red meat consumption and both iron status and metabolic health is critical. Using data from the Qatar Precision Health Institute—Qatar Biobank (QPHI-QBB), this study aims to investigate gender-specific associations between red meat intake, iron biomarkers (e.g., serum ferritin and hemoglobin), and metabolic profiles linked to cardiovascular and chronic diseases (e.g., lipoproteins, cholesterol, and inflammatory markers). By evaluating both the potential benefits of red meat for iron sufficiency and its associated health risks, this research seeks to inform culturally appropriate dietary guidelines that address iron deficiency anemia and chronic disease prevention in Qatar.

## 2. Methods

### 2.1. Data Source

This cross-sectional study employed quantitative data obtained from the Qatar Biobank (QBB), part of the Qatar Precision Health Initiative (QPHI-QBB). Access to the dataset was granted to researchers upon request through Qatar University, a key research partner in the QPHI-QBB collaboration. Initiated in 2012, the QPHI-QBB is an ongoing cohort study targeting the general population in Qatar. It includes Qatari nationals and long-term residents (≥15 years), aiming to support medical research both locally and globally. Participant recruitment has been conducted through various means, including online platforms, social networks (e.g., family and friends), and social media. By 2022, over 20,000 adults had been enrolled in the QBB study [16], which collects biological samples, imaging, and extensive health-related data. It also stores blood and urine samples in a state-of-the-art biorepository for long-term use [16,17]. The dataset used in this analysis contains comprehensive information on demographics, health status, biomarkers, and nutrition-related variables.

### 2.2. Ethical Considerations

Ethical approvals were obtained from the Institutional Review Boards (IRB) of both the QPHI-QBB (QF-QBB-RES-ACC-00207-0287) in April 2024 and Qatar University (QU-IRB 053/2025-EM) in March 2025. The original data were collected from participants voluntarily, with informed consent obtained from each participant in strict adherence to the IRB guidelines; no new consent was required to use the data in this study. The dataset has been shared with the researchers as de-identified data; no personal or sensitive information was included in the dataset, so the confidentiality and privacy of the participants were maintained.

### 2.3. Study Population

**Figure 1:**
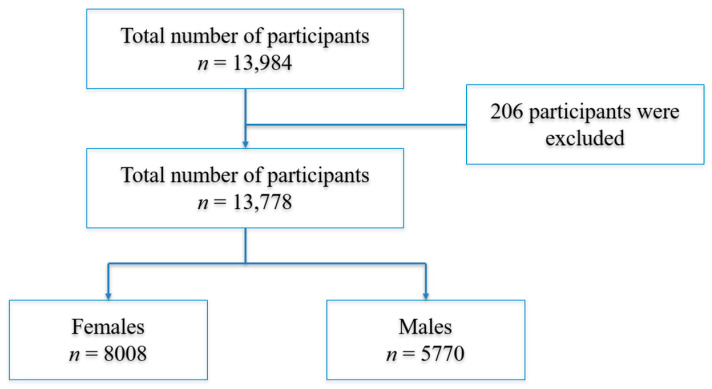
Study population selection after exclusion.

Data from 13,984 participants in the QPHI-QBB study were granted under project number QF-QBB-RES-ACC-00207. This dataset included nearly all participants enrolled at the time of data access. However, 206 individuals were excluded due to substantial missing data, resulting in a final analytic sample of 13,778 participants (men: *n* = 5770; women: *n* = 8008) (Figure 1). Eligibility criteria included Qatari adults aged 18 years and older. Participants were excluded if dietary intake records or biochemical marker data were missing. Health and lifestyle characteristics were obtained through self-reported questionnaires and clinical assessments recorded in the Qatar Biobank. Smoking status was categorized as follows: individuals who self-identified as current smokers were classified as smokers, whereas non-smokers included both former smokers and those who had never smoked. Diabetes was diagnosed based on self-reported physician diagnosis, use of glucose-lowering medications, or laboratory criteria (fasting plasma glucose ≥ 7.0 mmol/L or HbA1c ≥ 6.5%). High cholesterol was defined as self-reported diagnosis, use of lipid-lowering medications, or laboratory-measured total cholesterol ≥ 5.2 mmol/L. Hypertension was determined by self-reported diagnosis, antihypertensive medication use, or an average systolic/diastolic blood pressure ≥ 140/90 mmHg during Qatar Biobank health screenings.

### 2.4. Anthropometric Measurements

Trained nurses measured participants’ height and weight while they wore light clothing and no shoes, following standardized protocols established by the QPHI-QBB. Body weight and height were measured to the nearest 0.1 kg and 0.1 cm, respectively, by trained Qatar Biobank staff using calibrated digital scales and stadiometers, with participants wearing light clothing and no shoes, as part of standardized health assessments. Body mass index (BMI) was calculated as weight (kg) divided by height squared (m^2^). Measurements were conducted in a controlled environment at Qatar Biobank facilities, with participants fasting overnight to minimize variability. Further details on the QBB anthropometric measurement protocols are described by Al Thani et al. [16].

### 2.5. Biochemical Measurements

Venous blood samples were collected from participants after an overnight fast and sent to Hamad Medical Corporation Laboratories (accredited by the College of American Pathologists) for further analysis. Various blood biomarkers, including serum iron, total iron-binding capacity (TIBC), ferritin, serum 25(OH)D, plasma glucose, serum high-density lipoprotein (HDL) cholesterol, serum total cholesterol, serum low-density lipoprotein (LDL) cholesterol, serum triacylglycerol, C-reactive protein (CRP), insulin, folate, and vitamin B12, were measured simultaneously [16]. Iron levels were measured using a serum iron test with a colorimetric method. TIBC was assessed by saturating transferrin with iron and measuring the bound iron. Ferritin was measured using immunoassays (ELISA). Iron sufficiency was defined using WHO thresholds: hemoglobin (Hgb) ≥ 13 g/dL for males and ≥12 g/dL for females (sufficient), with Hgb < 13 g/dL for males and < 12 g/dL for females classified as deficient [18] Serum 25(OH)D levels, encompassing both vitamin D2 and D3 fractions, were determined using an electrochemiluminescence immunoassay (LIAISON^®^ 25-hydroxyvitamin D Total Assay, DiaSorin Inc., Stillwater, MN, USA). Vitamin D sufficiency was defined as serum 25(OH)D ≥ 20 ng/mL [19]. Serum total cholesterol was measured using the enzymatic was measured using the enzymatic cholesterol oxidase-phenol aminophenazone method (CHOD-PAP) method. The HDL-cholesterol Plus Third-Generation Method was used to measure serum HDL cholesterol, while the LDL-cholesterol Plus Second-Generation Method was used for serum LDL-cholesterol. Serum triacylglycerol was measured using the enzymatic glycerol-3-phosphate oxidase–phenol aminophenazone (GPO-PAP) method. CRP levels were determined using high-sensitivity immunoassays. Insulin levels were assessed using immunoassays such as radioimmunoassay (RIA) or ELISA. Glucose was measured using enzymatic methods with a glucometer. Folate and vitamin B12 levels were measured using immunoassays like ELISA or chemiluminescent immunoassay (CLIA) [20]. More details are mentioned in Al Kuwari et al. [20] and Al Thani et al. [16].

### 2.6. Dietary Assessment

The Qatar Biobank developed a culturally tailored, computer-administered dietary assessment tool based on local food assessments, focus groups, and expert consultation [16]. The Food Frequency Questionnaire (FFQ) assessed the intake of 102 food and beverage items using frequency-based responses and included additional questions on general dietary habits. Internal validity was evaluated by comparing broad food category responses with the sum of related individual items, yielding Spearman’s correlation coefficients ranging from 0.30 (for snacks) to 0.74 (for fish), indicating moderate to strong internal consistency [16]. Red meat intake was assessed across six frequency categories: never or rarely, 1–3 times per month, 1–3 times per week, 4–6 times per week, once per day, and two or more times per day. Data on supplementation were collected through questionnaires administered by QBB nurses. Participants were classified as taking supplementation if they had used iron, calcium, vitamin C, and folic acid supplements consistently for the preceding three months. The assessment of red meat consumption was based on the frequency of meat intake per month, as reported by participants.

### 2.7. Statistical Analysis

Statistical analyses were conducted using Stata 18.5 (StataCorp, College Station, TX, USA). Sociodemographic factors (employment status, education level, and smoking status) and supplement use (iron, calcium, vitamin C, and folic acid) were summarized using frequencies and percentages. Red meat consumption was originally captured using six frequency categories: never or rarely, 1–3 times/month, 1–3 times/week, 4–6 times/week, once/day, and ≥2 times/day. To improve analytical robustness and clarity, these were collapsed into three groups: low (≤1 time/month), moderate (2–4 times/month), and high (≥5 times/month). These thresholds were selected to capture clinically relevant distinctions in intake frequency, ensure consistency with the Food Frequency Questionnaire’s framework, and reflect population-specific intake patterns observed in Qatar. Continuous variables (e.g., age, body mass index, hemoglobin, ferritin, and cholesterol) were reported as means and standard deviations, stratified by low, moderate, and high red meat consumption categories. Stratification was further performed by gender (males, females). Two-sample *t*-tests compared continuous variables (total cholesterol, high-density lipoprotein cholesterol, triglycerides, glucose, insulin, folate, vitamin B12, 25-hydroxyvitamin D, and C-reactive protein) between genders within each red meat consumption category. One-way analysis of variance (ANOVA) assessed differences in hematological markers (hemoglobin, ferritin, TIBC, iron) across red meat consumption categories, adjusted for age and gender. Post-hoc pairwise comparisons were conducted with Bonferroni correction to explore significant ANOVA differences across red meat consumption categories (Low, Moderate, High) within each gender. Comparisons (Low vs. Moderate, Low vs. High, Moderate vs. High) were evaluated for each parameter per gender, with significance set at a Bonferroni-adjusted *p* < 0.05.

Multiple linear regression models examined associations between red meat consumption and hematological markers, adjusting for gender, age, supplement use, diabetes, high cholesterol, and high blood pressure, with interaction terms for red meat consumption, gender, and age. Statistical significance was set at *p* ≤ 0.05.

## 3. Results

### 3.1. Baseline Characteristics of Study Participants

The study included a total of 13,778 participants, and data were obtained from the QBB database, with 5770 males (41.8%) and 8008 females (58%). Table 1 shows that males had a lower mean BMI (29.5 ± 5.1 kg/m^2^) compared to females (31.9 ± 6.0 kg/m^2^, *p* < 0.0001) and were slightly younger (47.9 ± 9.7 years vs. 48.5 ± 9.5 years, *p* = 0.0003). Across red meat consumption categories, mean BMI (30.8–30.9 kg/m^2^) and age (48.2–48.5 years) showed no significant differences (*p* = 0.9499 for BMI, *p* = 0.5945 for age). Employment status indicated that 81.6% of males (*n* = 4455) and 49.41% of females (*n* = 3888) were employed, with no significant variation across red meat consumption categories (*p* = 0.896 for males, *p* = 0.928 for females). Education levels differed by gender, with 49.9% of males (*n* = 2819) and 44.0% of females (*n* = 3516) holding university or postgraduate qualifications (*p* < 0.0001), and higher illiteracy rates among females (9.3%, *n* = 740) compared to males (3.1%, *n* = 180, *p* < 0.0001). Education distribution across red meat consumption categories was consistent (*p* = 0.983 for university/postgraduate, *p* = 0.536 for illiteracy). Smoking prevalence was higher in males (30%, *n* = 1733) than in females (25%, *n* = 2000, *p* < 0.0001), with no significant difference across red meat consumption categories (*p* = 0.319). Medical conditions showed diabetes in 23.9% of males (*n* = 1365) and 27.9% of females (*n* = 2227, *p* < 0.0001), high cholesterol in 40.1% of males (*n* = 2297) and 36.2% of females (*n* = 2890, *p* < 0.0001), and high blood pressure in 22.2% of males (*n* = 1262) and 21.9% of females (*n* = 1749, *p* = 0.845), with no significant variation by red meat consumption (*p* = 0.138, 0.844, 0.395, respectively). Supplementation was more common among females, with 21.7% using iron (vs. 4.6% males, *p* < 0.0001), 10.0% using calcium (vs. 4.1%, *p* < 0.0001), and 8.4% using vitamin C (vs. 6.8%, *p* = 0.0010). Across red meat consumption categories, supplementation rates were generally stable, except for vitamin C, which was higher in the high red meat consumption group (8.2%) compared to the low red meat consumption group (6.1%, *p* = 0.0280) (Table 1).

Table 2 presents biomarker levels by iron supplementation status (14.5%, *n* = 1997). Participants using iron supplements had slightly lower TIBC (67.2 ± 11.7 µg/dL vs. 67.8 ± 11.8 µg/dL, *p* = 0.0355), higher ferritin (94.6 ± 117.2 ng/mL vs. 88.9 ± 120.2 ng/mL, *p* = 0.0456), and significantly lower hemoglobin (12.4 ± 1.6 g/dL vs. 13.6 ± 1.7 g/dL, *p* < 0.0001) compared to those not using supplements. Serum iron levels were slightly higher in the supplementation group (14.9 ± 6.3 µg/dL vs. 14.7 ± 6.4 µg/dL), but the difference was not statistically significant (*p* = 0.1598).

### 3.2. Hematological Parameters Across Red Meat Consumption Levels

A detailed analysis of hematological parameters across males and females, categorized by red meat consumption levels (low, moderate, high), revealed distinct patterns. Table 3 shows that Hgb levels were consistently higher in males across all red meat consumption categories (low: 14.8 ± 1.3 g/dL; moderate: 14.9 ± 1.2 g/dL; high: 14.9 ± 1.3 g/dL) compared to females (low: 12.5 ± 1.4 g/dL; moderate: 12.5 ± 1.3 g/dL; high: 12.4 ± 1.3 g/dL), with statistically significant differences (*p* < 0.0001 for all categories). TIBC levels showed no significant gender differences (low: 67.7 ± 11.6 µmol/L in males vs. 68.6 ± 12.3 µmol/L in females, *p* = 0.1775; moderate: 67.8 ± 11.7 µmol/L vs. 68.1 ± 11.8 µmol/L, *p* = 0.1590; High: 64.8 ± 11.4 µmol/L vs. 65.1 ± 11.4 µmol/L, *p* = 0.6558). Ferritin levels were elevated in the high red meat consumption group for both genders (males: 218.1 ± 293.4 µg/L; females: 214.3 ± 257.8 µg/L, *p* = 0.8037), with no significant gender differences at low and moderate levels (low: 72.3 ± 78.3 µg/L in males vs. 76.2 ± 81.0 µg/L in females, *p* = 0.3422; Moderate: 76.2 ± 77.9 µg/L vs. 77.1 ± 79.2 µg/L, *p* = 0.5232). Serum iron levels showed no significant gender differences (*p* > 0.5), with higher values in the high red meat consumption group (males: 16.0 ± 6.7 µg/dL, females: 16.3 ± 7.4 µg/dL) compared to the low (14.2–14.4 µg/dL) and moderate (14.6 ± 6.3–6.4 µg/dL) red meat consumption groups. Serum iron levels showed no significant gender differences (*p* > 0.5), with higher values in the high red meat consumption group (males: 16.0 ± 6.7 µg/dL, females: 16.3 ± 7.4 µg/dL) compared to the low (14.2–14.4 µg/dL) and moderate (14.6 ± 6.3–6.4 µg/dL) meat consumption groups.

The analysis revealed significant gender disparities in Hgb levels across all red meat consumption categories (Low, Moderate, High: *p*< 0.0001 for all), with males consistently exhibiting higher values (14.8–14.9 g/dL) compared to females (12.4–12.5 g/dL). However, Hgb levels did not vary significantly with meat consumption intensity for either gender (*p* = 0.8955 for males; *p* = 0.6939 for females). TIBC decreased significantly with higher red meat intake (*p* < 0.0001 for both genders), with the high red meat consumption group showing the lowest values (males: 64.8 ± 11.4 µmol/L; females: 65.1 ± 11.4 µmol/L). Ferritin levels exhibited a marked increase with higher red meat consumption (*p* < 0.0001 for both genders), rising from ~72–77 µg/L in the low/moderate red meat consumption groups to ~214–218 µg/L in the high red meat consumption group. Similarly, serum iron levels significantly increased with red meat consumption intensity (*p* < 0.0001 for both genders), peaking in the high category (males: 16.0 ± 6.7 µg/dL; females: 16.3 ± 7.4 µg/dL). These findings suggest that red meat consumption intensity strongly influences iron-related biomarkers, independent of gender, while Hgb differences remain gender-specific and unaffected by intake levels.

### 3.3. Metabolic Parameters and Vitamin Levels Across Red Meat Consumption Levels

Following the analysis of hematological parameters, the study examined metabolic and vitamin markers across low (≤1 time/month), moderate (2–4 times/month), and high (≥5 times/month) red meat consumption categories, stratified by gender, to assess their associations with dietary intake, as shown in Table 4. Among cardiometabolic markers, total cholesterol was significantly higher in males within the high red meat consumption group (5.2 ± 1.1 mmol/L) compared to females (5.0 ± 1.0 mmol/L, *p* = 0.0125), suggesting a gender-specific difference at elevated red meat intake levels. Similarly, LDL-cholesterol showed a significant gender difference in the high red meat consumption group, with males having higher levels (3.1 ± 1.0 mmol/L) than females (2.9 ± 0.9 mmol/L, *p* = 0.0008), but no notable differences in the low (males: 3.0 ± 0.9 mmol/L, females: 3.0 ± 0.8 mmol/L, *p* = 0.1602) or moderate (males: 3.0 ± 0.9 mmol/L, females: 3.0 ± 0.9 mmol/L, *p* = 0.5160) categories. Folate levels exhibited a borderline gender difference in the moderate red meat consumption group, with females having slightly higher levels (24.6 ± 10.9 ng/mL) than males (24.2 ± 10.7 ng/mL, *p* = 0.0929), a finding potentially linked to its role in heme synthesis. Other parameters, including HDL cholesterol, triglycerides, glucose, insulin, and vitamin B12, showed no notable gender differences (*p* > 0.0566). Vitamin D levels, relevant to overall health and iron metabolism interactions, were comparable across categories (e.g., moderate group: 23.6 ± 12.5 ng/mL in males vs. 23.2 ± 12.6 ng/mL in females, *p* = 0.1191). CRP levels, a marker of inflammation pertinent to ferritin assessment, showed no significant gender differences (e.g., moderate group: 5.7 ± 6.2 mg/L in males vs. 5.9 ± 7.1 mg/L in females, *p* = 0.1883; high group: 5.8 ± 7.5 mg/L vs. 5.9 ± 7.8 mg/L, *p* = 0.8173).

The analysis revealed significant associations between red meat consumption levels and specific biomarkers, stratified by gender. In males, higher red meat consumption significantly influenced HDL (*p* = 0.0004), triglycerides (*p* = 0.0070), LDL (*p* = 0.0120), and glucose (*p* = 0.0277). Among females, significant effects were observed for HDL (*p* = 0.0031), triglycerides (*p* = 0.0179), glucose (*p* < 0.0001), and vitamin B12 (*p* = 0.0025). Notably, LDL and vitamin B12 exhibited gender-specific disparities, with LDL significance limited to males and vitamin B12 to females. Conversely, no significant differences were detected for cholesterol, insulin, folate, vitamin D, or CRP in either gender (*p* > 0.05 for all).

### 3.4. Adjusted Associations of Red Meat Consumption with Hematological Parameters

To further explore the relationships observed in hematological and metabolic parameters, multiple linear regression models were used to assess the associations between red meat consumption and hematological markers (hemoglobin, ferritin, total iron-binding capacity [TIBC], and iron), as shown in Table 5. Models were adjusted for gender, age, supplement use (iron, calcium, vitamin C, and folic acid), diabetes, high cholesterol, high blood pressure, and education, with interaction terms for meat consumption, gender, and age included to capture potential effect modifications. High red meat consumption was strongly associated with elevated ferritin levels compared to low red meat consumption (Coef = 134.685, *p* < 0.001), with a significant age-related decline in ferritin among females with high red meat intake (High × Female × Age: Coef = −1.957, *p* = 0.031). Hemoglobin showed a strong association with gender (Female vs. Male: Coef = −4.646, *p* < 0.001) and a modest association with high red meat consumption (High vs. Low: Coef = 0.918, *p* = 0.017). Additionally, hemoglobin was influenced by age (Coef = −0.014, *p* = 0.007) and supplement use, notably iron (Coef = −0.456, *p* < 0.001) and vitamin C (Coef = 0.146, *p* = 0.001). By contrast, TIBC (High vs. Low: Coef = −1.527, *p* = 0.670) and iron (High vs. Low: Coef = 0.045, *p* = 0.982) showed no significant associations with high red meat consumption, nor did age significantly affect TIBC (Coef = 0.025, *p* = 0.617) or iron (Coef = −0.008, *p* = 0.760). These findings highlight the complex interplay of dietary and health-related factors in hematological outcomes.

## 4. Discussion

The present study aimed to investigate the potential association between red meat consumption, iron status, and metabolic health. The study findings showed that higher red meat consumption significantly enhances ferritin levels, a key indicator of iron stores, reflecting the superior bioavailability of heme iron compared to non-heme sources [1]. Heme iron, absorbed via specific intestinal transporters (e.g., heme carrier protein 1), bypasses the regulatory constraints of non-heme iron absorption, which is modulated by hepcidin and dietary inhibitors like phytates [21]. This mechanism explains the robust ferritin increase observed with frequent meat intake, consistent with studies in diverse populations [22,23]. In females, ferritin levels decline with age, particularly at higher consumption levels, likely due to reduced iron demands post-menopause, as estrogen modulates hepcidin expression, affecting iron homeostasis [6,24]. Hemoglobin, essential for oxygen transport, was consistently higher in males, a finding attributable to androgen-driven erythropoiesis and lower iron losses compared to females, who face menstrual blood loss [3,25]. These gender differences underscore the need for tailored dietary strategies to address iron deficiency, especially in females.

The study also revealed that TIBC and serum iron are less responsive to red meat intake compared to ferritin. TIBC, which reflects transferrin’s capacity to bind iron, was elevated in females at lower red meat consumption levels, indicating a compensatory response to higher iron needs [26]. This aligns with the physiological upregulation of transferrin synthesis in iron-deficient states, mediated by hypoxia-inducible factors [27]. However, neither TIBC nor serum iron showed significant dietary associations, likely due to tight homeostatic regulation by hepcidin, which limits intestinal iron absorption and systemic iron release [28,29]. These findings highlight ferritin’s reliability as a long-term biomarker for dietary iron interventions, as serum iron fluctuates daily and TIBC responds more to deficiency than dietary excess [30].

Despite significant gender disparities in Hgb levels, with males exhibiting consistently higher values (14.8–14.9 g/dL) than females (12.4–12.5 g/dL) across all red meat intake categories (*p* < 0.0001), Hgb remained unaffected by dietary intensity in either gender (*p* > 0.05). This reflects inherent biological differences, such as androgen-driven erythropoiesis in males and menstrual iron loss in females, which dominate over dietary influences [31]. Conversely, red meat consumption strongly modulated iron metabolism biomarkers in a gender-neutral manner: TIBC decreased (*p* < 0.0001), while ferritin and serum iron levels increased with higher red meat intake, doubling in the high red meat consumption group (*p* < 0.0001). These changes underscore the superior bioavailability of heme iron from meat, which enhances iron absorption and storage without altering gender-specific Hgb baselines [32]. The divergence between static Hgb and dynamic iron stores suggests distinct regulatory mechanisms, where Hgb is tightly controlled by physiological priorities (e.g., oxygen transport), whereas ferritin and TIBC reflect dietary adaptability [33].

Metabolic outcomes, particularly lipid profiles, revealed gender-specific patterns linked to red meat consumption. Males exhibited higher total cholesterol levels at elevated red meat intake levels, reflecting the impact of dietary saturated fats and cholesterol in red meat on hepatic LDL receptor activity [11,34]. Females, conversely, showed higher HDL cholesterol, which may mitigate cardiovascular risk by enhancing reverse cholesterol transport [35,36]. In a study by Kim and Shin, red meat consumption was associated with 46 metabolites, including various lipoproteins, fatty acids, and phospholipids, 29 of which were also linked to increased CVD risk [37]. While their analysis was based on a metabolomics panel, our findings reinforce this relationship using clinical biomarkers, showing that higher red meat intake was associated with elevated ferritin, hemoglobin, and total cholesterol, particularly in men. This suggests that although the two studies differ in methodology and outcome measures, both highlight a consistent metabolic impact of red meat consumption with potential implications for cardiometabolic health. The absence of significant gender differences in triglycerides, glucose, and insulin suggests that the metabolic impact of red meat at the studied frequencies (up to ≥5 times/month) is primarily lipid-related, consistent with moderate intake posing limited risk in other populations. A systematic review and meta-analysis of cohort studies found that the associations between red and processed meat consumption and all-cause mortality or adverse cardiometabolic outcomes were modest in magnitude, with the overall certainty of evidence rated as low [38].

Vitamins and inflammation markers provided further context for iron metabolism. Folate, marginally higher in females at moderate consumption, supports heme synthesis by facilitating tetrahydrofolate-dependent nucleotide production [25,39]. Vitamin D levels, sufficient in both genders at moderate red meat intake, may reflect biofortification in Qatari livestock, enhancing meat’s nutritional value [40,41]. CRP, an inflammation marker, showed no gender differences, indicating that systemic inflammation does not significantly influence ferritin levels in this cohort, unlike in conditions with chronic inflammation [25,42]. This dissociation strengthens the interpretation of ferritin as a dietary iron marker rather than an acute-phase reactant in healthy Qatari adults.

The observed gender-specific metabolic responses to red meat consumption align with existing evidence on sex differences in lipid and glucose metabolism. For instance, males exhibited significant alterations in HDL, triglycerides, LDL, and glucose, which may reflect hormonal influences (e.g., testosterone’s role in lipid partitioning) or differences in body composition [43]. The absence of associations between red meat intake and cholesterol, insulin, or CRP contrasts with prior reports linking processed meats (e.g., sausages and bacon) to dyslipidemia and inflammation, as seen in meta-analyses by O’Connor et al. [44]. This discrepancy suggests that unprocessed meats (e.g., lean cuts and poultry), which lack additives like nitrates and excess sodium, may exert neutral or context-dependent metabolic effects. Furthermore, confounding factors such as concurrent fiber intake, common in diets rich in unprocessed meats paired with vegetables, could attenuate adverse lipid or inflammatory responses, as fiber mitigates postprandial lipidemia and modulates gut-mediated inflammation [45]. These findings underscore the need to differentiate meat types and contextualize dietary patterns when evaluating cardiometabolic risks. The pronounced sensitivity of females to glucose regulation and vitamin B12 levels observed in this study may reflect estrogen’s role in enhancing insulin signaling pathways, as demonstrated in experimental models where estrogen receptor activation improves pancreatic β-cell function and peripheral glucose uptake [46]. Gender-specific dietary patterns, such as lower red meat consumption and higher reliance on plant-based or fortified foods among females, could explain the vulnerability to B12 variability. For instance, studies suggest that women, particularly those adhering to vegetarian or low-meat diets, are at higher risk of B12 deficiency due to reduced intake of bioavailable animal-derived B12 [47].

Supplementation, particularly with iron and vitamin C, significantly influenced iron status [48]. Iron supplementation, often targeted at deficient individuals, and vitamin C, which enhances non-heme iron absorption via ferric-to-ferrous reduction, modulated hemoglobin levels [30,49]. Age also played a role, affecting iron storage (ferritin) and erythropoiesis (hemoglobin), likely due to changes in hepcidin regulation and red blood cell turnover [50,51]. These findings highlight the synergistic effects of dietary and supplemental iron sources, particularly for females, in optimizing iron status.

The findings of this study carry important public health implications for Qatar, where lamb is a culturally significant dietary staple. Moderate red meat consumption (2–4 times/month) enhances iron status, particularly ferritin, without notable metabolic risks, providing a balanced strategy to combat iron deficiency, especially in females with higher iron requirements due to physiological losses. However, more frequent intake (≥5 times/month) necessitates cholesterol monitoring in males to address potential cardiovascular risks. Studies in Middle Eastern countries, such as Saudi Arabia and the United Arab Emirates, with comparable dietary patterns emphasizing lamb and similar preparation methods (e.g., grilling and stewing), have reported parallel findings, reinforcing the positive association between red meat intake and iron status [52,53]. For instance, research in Saudi Arabia has shown increased ferritin levels with regular red meat consumption, alongside gender differences in hemoglobin, mirroring the male-female disparities observed here [54]. These studies validate the potential of moderate red meat intake to improve iron profiles while highlighting the need for lipid surveillance in males. Future longitudinal research in the region should explore causal pathways, incorporating genetic factors like hepcidin gene variants, which may influence iron absorption, and lifestyle factors, such as cooking techniques that affect heme iron bioavailability [11,32,55]. Such investigations could confirm the generalizability of these findings across Middle Eastern populations and inform tailored dietary guidelines to optimize iron and metabolic health.

## 5. Study Limitations and Strengths

This is the first study to investigate the association between red meat consumption, iron profiles, and other metabolomic parameters in the Qatari population. The study has several notable strengths. The first strength is the use of the comprehensive FFQ, which included a diverse range of 102 foods commonly consumed in Qatar. Despite the study’s strengths, it has a few limitations. First, dietary intake was assessed using the FFQ, which is subject to recall bias and may not accurately capture habitual consumption. Second, residual confounding is possible, as comorbidities such as diabetes, hypertension, and hypercholesterolemia were self-reported and may be under- or overestimated. Another limitation is the cross-sectional design, which makes it challenging to establish temporality or causality. Additionally, although the sample size is large, the findings are limited to the characteristics of the volunteers in this dataset, making it challenging to generalize the results to the broader Qatari population. This study has several limitations that should be acknowledged. Lastly, the analysis did not account for portion sizes or differentiate between types of red meat (e.g., lean vs. processed), which may have varying effects on iron status and metabolic outcomes.

## 6. Conclusions

This study demonstrates that red meat consumption significantly enhances iron status among the Qatari population. Frequent intake is associated with a marked increase in ferritin levels and a modest improvement in hemoglobin, particularly among males, who exhibit higher hemoglobin concentrations than females. By contrast, females show age-related declines in ferritin despite higher consumption. Total iron-binding capacity and serum iron appear to be largely unaffected by red meat intake, highlighting ferritin as the most reliable biomarker of iron status. Notably, frequent consumption in males is linked to elevated cholesterol levels, whereas other metabolic parameters, such as glucose and triglycerides, show minimal dietary influence. Vitamin D levels remain sufficient across all consumption categories. These findings support public health recommendations in Qatar to encourage moderate red meat intake (2–4 times per month) to improve iron status—especially in females at risk of deficiency—while advising cholesterol monitoring in males to mitigate cardiovascular risks. Further longitudinal studies are warranted to assess long-term dietary effects and inform tailored nutritional guidelines for Qatar and similar populations.

## Figures and Tables

**Table 1:** Baseline characteristics of study participants by gender and red meat consumption categories.

Characteristic	Gender		*p*-Value (Gender)	Red Meat Consumption			*p*-Value (Meat)
	Males (*n* = 5760)	Females (*n* = 7987)		Low (*n* = 1612)	Moderate (*n* = 10,809)	High (*n* = 1326)	
Age (years, mean ± SD)	47.9 ± 9.7	48.5 ± 9.5	0.0003 *	48.3 ± 9.6	48.2 ± 9.7	48.5 ± 9.5	0.5945 **
BMI (kg/m^2^, mean ± SD)	29.5 ± 5.1	31.9 ± 6.0	<0.0001 *	30.8 ± 5.8	30.8 ± 5.8	30.9 ± 5.6	0.9499 **
% (*n*)							
Employment Status							
- Employed (Males) ^1^	81.6 (4455)	-	-	81.3 (523)	81.7 (3500)	81.6 (432)	0.8960 ***
- Employed (Females) ^1^	-	49.4 (3888)	-	49.1 (472)	49.5 (3020)	49.2 (396)	0.9280 ***
Education			<0.0001 ***				0.9830 ***
- University/Postgraduate	49.9 (2819)	44.0 (3516)		45.3 (730)	46.6 (5032)	45.8 (605)	
- Illiterate	3.1 (180)	9.3 (740)		6.8 (110)	6.7 (724)	6.5 (86)	0.5360 ***
Smoking Status			<0.0001 ***				0.3190 ***
- Smoker ^2^	30.0 (1733)	25.0 (2000)		71.2 (1147)	70.2 (7585)	71.1 (940)	
Medical Conditions							
- Diabetes ^3^	23.9 (1365)	27.9 (2227)	<0.0001 ***	27.8 (446)	25.8 (2783)	27.5 (363)	0.1380 ***
- High Cholesterol ^4^	40.1 (2297)	36.2 (2890)	<0.0001 ***	38.4 (616)	37.7 (4066)	38.3 (505)	0.8440 ***
- High Blood Pressure ^5^	22.2 (1262)	21.9 (1749)	0.8450 ***	20.8 (334)	22.0 (2376)	22.8 (301)	0.3950 ***
Supplementation Status							
- Iron Supplementation ^6^	4.6 (266)	21.7 (1731)	<0.0001 ***	14.8 (239)	14.4 (1562)	14.8 (196)	0.8890 ***
- Calcium Supplementation ^7^	4.1 (236)	10.0 (796)	<0.0001 ***	7.3 (118)	7.6 (819)	7.2 (95)	0.8260 ***
- Vitamin C Supplementation ^8^	6.8 (393)	8.4 (670)	0.0010 ***	6.1 (98)	7.9 (856)	8.2 (109)	0.0280 ***

* *p*-value from two-sample *t*-test. ** *p*-value from one-way ANOVA. *** *p*-value from chi-squared test. ^1^ Employed: Employment male = 1 for males, Employment female = 1 for females. ^2^ Smoker: Current smoker (self-reported). ^3^ Diabetes: Diagnosed with diabetes (self-reported or clinical diagnosis). ^4^ High Cholesterol: Diagnosed with high cholesterol (self-reported or clinical diagnosis). ^5^ High Blood Pressure: Diagnosed with hypertension (self-reported or clinical diagnosis). ^6^ Iron Supplementation: Self-reported use of iron supplements for ≥3 months. ^7^ Calcium Supplementation: Self-reported use of calcium supplements for ≥3 months. ^8^ Vitamin C Supplementation: Self-reported use of vitamin C supplements for ≥3 months.

**Table 2:** Iron supplementation status and mean biomarker levels.

Biomarker	No Supplementation (*n* = 11,750, 85.5%)	Iron Supplementation (*n* = 1997, 14.5%)	*p*-Value
TIBC (µg/dL)	67.8 ± 11.8	67.2 ± 11.7	0.0355
Iron (µg/dL)	14.7 ± 6.4	14.9 ± 6.3	0.1598
Ferritin (ng/mL)	88.9 ± 120.2	94.6 ± 117.2	0.0456
Hgb (g/dL)	13.6 ± 1.7	12.4 ± 1.6	<0.0001

**Abbreviation**: Hgb: Hemoglobin; TIBC: Total iron-binding capacity.

**Table 3:** Hematological parameters across red meat consumption levels by gender.

Parameter	Red Meat Consumption	Males (Mean ± SD)	Females (Mean ± SD)	*p*-Value * (Gender)
Hgb (g/dL)	Low	14.8 ± 1.2 ^a^	12.4 ± 1.4 ^a^	<0.0001
	Moderate	14.9 ± 1.2 ^a^	12.5 ± 1.3 ^a^	<0.0001
	High	14.8 ± 1.3 ^a^	12.4 ± 1.3 ^a^	<0.0001
	*p*-value **	0.8955	0.6939	
TIBC (µmol/L)	Low	67.7 ± 11.6 ^a^	68.6 ± 12.3 ^a^	0.1775
	Moderate	67.8 ± 11.7 ^a^	68.1 ± 11.8 ^a^	0.1590
	High	64.9 ± 11.4 ^b^	65.1 ± 11.4 ^b^	0.6558
	*p*-value **	0.0001	0.0001	
Ferritin (µg/L)	Low	72.3 ± 78.3 ^a^	76.2 ± 81.0 ^a^	0.3422
	Moderate	76.2 ± 77.9 ^a^	77.1 ± 79.2 ^a^	0.5232
	High	218.1 ± 293.4 ^b^	214.3 ± 257.8 ^b^	0.8037
	*p*-value **	0.0001	0.0001	
Iron (µg/dL)	Low	14.2 ± 6.1 ^a^	14.4 ± 6.0 ^a^	0.5980
	Moderate	14.6 ± 6.3 ^a^	14.6 ± 6.4 ^a^	0.6387
	High	16.0 ± 6.7 ^b^	16.3 ± 7.4 ^b^	0.5405
	*p*-value **	0.0001	0.0001	

* *p*-value from two-sample *t*-test comparing males and females within each red meat consumption category. ** *p*-value from ANOVA post hoc tests: Different letters indicate statistically significant differences between red meat consumption categories. Meat consumption categories: Low (≤1 time/month), Moderate (2–4 times/month), High (≥5 times/month). **Abbreviation**: Hemoglobin (Hgb); Total iron-binding capacity (TIBC).

**Table 4:** Metabolic parameters and vitamins across red meat consumption levels by gender.

Parameter	Red Meat Consumption	Males (Mean ± SD)	Females (Mean ± SD)	*p*-Value * (Gender)
Cholesterol (mmol/L)	Low	5.1 ± 1.0 ^a^	5.0 ± 0.9 ^a^	0.1013
	Moderate	5.1 ± 1.0 ^a^	5.1 ± 1.0 ^a^	0.5421
	High	5.2 ± 1.1 ^a^	5.0 ± 1.0 ^a^	0.0125
	*p*-value **	0.0963	0.1358	
HDL (mmol/L)	Low	1.4 ± 0.4 ^a^	1.4 ± 0.4 ^a^	0.3703
	Moderate	1.4 ± 0.4 ^a^	1.4 ± 0.4 ^a^	0.5318
	High	1.4 ± 0.4 ^b^	1.4 ± 0.4 ^b^	0.3987
	*p*-value **	0.0004	0.0031	
Triglyceride (mmol/L)	Low	1.4 ± 0.8 ^a^	1.4 ± 0.7 ^a^	0.9749
	Moderate	1.4 ± 0.8 ^a^	1.4 ± 0.8 ^a^	0.4093
	High	1.5 ± 1.0 ^b^	1.5 ± 0.9 ^b^	0.4294
	*p*-value **	0.0070	0.0179	
LDL (mmol/L)	Low	3.0 ± 0.9 ^a^	3.0 ± 0.8 ^a^	0.1602
	Moderate	3.0 ± 0.9 ^a^	3.0 ± 0.9 ^a^	0.5160
	High	3.1 ± 1.0 ^b^	2.9 ± 0.9 ^a^	0.0008
	*p*-value **	0.0120	0.1240	
Glucose (mmol/L)	Low	6.0 ± 2.8 ^a^	5.9 ± 2.3 ^a^	0.3557
	Moderate	5.9 ± 2.2 ^ab^	6.0 ± 2.5 ^a^	0.0566
	High	6.2 ± 2.6 ^ac^	6.4 ± 3.0 ^b^	0.0897
	*p*-value **	0.0277	0.0001	
Insulin (µIU/mL)	Low	14.4 ± 14.6 ^a^	13.6 ± 12.2 ^a^	0.2353
	Moderate	14.1 ± 15.9 ^a^	14.8 ± 33.9 ^a^	0.1438
	High	14.5 ± 14.3 ^a^	14.8 ± 13.2 ^a^	0.6827
	*p*-value **	0.7405	0.5028	
Folate (ng/mL)	Low	24.2 ± 10.9 ^a^	24.4 ± 11.4 ^a^	0.7685
	Moderate	24.2 ± 10.7 ^a^	24.6 ± 10.9 ^a^	0.0929
	High	24.1 ± 11.0 ^a^	24.5 ± 11.0 ^a^	0.5060
	*p*-value **	0.9530	0.9052	
Vitamin B12 (pg/mL)	Low	312.0 ± 145.9 ^a^	309.9 ± 158.7 ^a^	0.7895
	Moderate	311.3 ± 158.4 ^a^	309.8 ± 156.2 ^a^	0.6241
	High	328.5 ± 159.2 ^a^	330.4 ± 155.0 ^b^	0.8254
	*p*-value **	0.0566	0.0025	
Vitamin D (ng/mL)	Low	23.6 ± 12.7 ^a^	23.8 ± 12.5 ^a^	0.7383
	Moderate	23.6 ± 12.5 ^a^	23.2 ± 12.6 ^a^	0.1191
	High	22.6 ± 12.4 ^a^	22.8 ± 11.9 ^a^	0.6032
	*p*-value **	0.2228	0.2369	
CRP (mg/L)	Low	6.0 ± 6.6 ^a^	5.8 ± 6.9 ^a^	0.5160
	Moderate	5.7 ± 6.2 ^a^	5.9 ± 7.1 ^a^	0.1883
	High	5.8 ± 7.5 ^a^	5.9 ± 7.8 ^a^	0.8173
	*p*-value **	0.6469	0.8039	

* *p*-value from two-sample *t*-test comparing males and females within each red meat consumption category. ** *p*-value from ANOVA post hoc tests: Different letters indicate statistically significant differences between red meat consumption categories. Meat consumption categories: Low (≤ 1 time/month), Moderate (2–4 times/month), High (≥5 times/month).

**Table 5:** Adjusted associations of red meat consumption with hematological parameters.

Predictor	Hgb Coef	Hgb *p*-Value	Ferritin Coef	Ferritin *p*-Value	TIBC Coef	TIBC *p*-Value	Iron Coef	Iron *p*-Value
Moderate Meat	0.017	0.949	10.097	0.676	3.486	0.168	−1.577	0.251
High Meat	0.918 *	0.017	134.685 ***	0.000	−1.527	0.670	0.045	0.982
Female	−4.646 ***	0.000	−5.840	0.843	4.601	0.135	0.319	0.849
Moderate × Female	0.162	0.645	12.678	0.687	−4.118	0.210	−0.246	0.890
High × Female	−0.806	0.104	86.533	0.051	−4.762	0.303	−0.437	0.862
Age	−0.014 **	0.007	0.809	0.084	0.025	0.617	−0.008	0.760
Moderate × Age	0.000	0.998	−0.144	0.772	−0.070	0.180	0.040	0.156
High × Age	−0.019 *	0.015	0.249	0.725	−0.026	0.724	0.035	0.381
Female × Age	0.048 ***	0.000	0.235	0.695	−0.077	0.221	−0.003	0.919
Moderate × Female × Age	−0.003	0.641	−0.312	0.626	0.074	0.270	0.001	0.980
High × Female × Age	0.016	0.112	−1.957 *	0.031	0.084	0.371	0.011	0.833
Iron Supplementation	−0.456 ***	0.000	1.231	0.692	−0.418	0.198	0.082	0.642
Calcium Supplementation	0.135 **	0.002	−12.366 **	0.002	0.624	0.133	−0.61 **	0.007
Vitamin C Supplementation	0.146 ***	0.001	23.354 ***	0.000	−2.11 ***	0.000	0.90 ***	0.000
Folic Acid	0.040	0.545	2.120	0.720	−0.041	0.947	0.644	0.055
Diabetes	−0.158 ***	0.000	−3.180	0.198	0.284	0.270	−0.088	0.532
High Cholesterol	0.112 ***	0.000	−7.168 ***	0.001	0.717 **	0.002	−0.35 **	0.005
High Blood Pressure	−0.151 ***	0.000	−7.612 **	0.003	0.375	0.163	−0.209	0.154
Constant	15.422 ***	0.000	14.921	0.518	66.95 ***	0.000	14.35 ***	0.000
Observations	13,685		13,680		13,694		13,690	
R-squared	0.487		0.128		0.011		0.010	

Multiple linear regression models; Coefficients with *p*-values. Coefficient: * *p* < 0.05, ** *p* < 0.01, *** *p* < 0.001. Supplementary Figures S1 and S2 provide box plots illustrating the distribution of ferritin and TIBC levels across red meat consumption categories, stratified by gender.

## Data Availability

The original contributions presented in the study are included in the article/Supplementary Materials, further inquiries can be directed to the corresponding author.

## Supplementary Materials

The following supporting information can be downloaded at: https://www.mdpi.com/article/10.3390/foods14122134/s1, Figure S1: Boxplot of ferritin levels across red meat consumption categories by gender. Figure S2: Boxplot of TIBC levels across red meat consumption categories by gender.
