# Supplementary material for: Red Meat Consumption, Iron Status, and Cardiometabolic Risk in Qatari Adults: A Cross-Sectional Gender-Stratified Analysis from the QPHI-QBB Data in Qatar
Source: Foods. 2025 Jun 18;14(12):2134. doi: 10.3390/foods14122134 (PMC12191705; doi:10.3390/foods14122134)
Supplement: Supplementary file 1 [file foods-14-02134-s001.zip › foods-3569143-supplementary.pdf]

Supplementary Materials

Ferritin levels were similar between genders (p>0.3), but markedly higher in the High meat consumption group (males: 218.1 ± 293.4 µg/L, females: 214.3 ± 257.8 µg/L) compared to Low and Moderate groups (72.3–77.1 µg/L). Ferritin levels in the High meat consumption group showed high variability (SD > 257 µg/L), with means of 218.1 µg/L for males and 214.3 µg/L for females, and no significant gender difference (p=0.8081). TIBC levels in the High meat consumption group exhibited moderate variability (SD ~ 11.4 µmol/L), with means of 64.8 µmol/L for males and 65.1 µmol/L for females, showing no significant gender difference (p=0.6558). Serum iron levels showed no significant gender differences (p>0.5), with higher values in the High meat group.

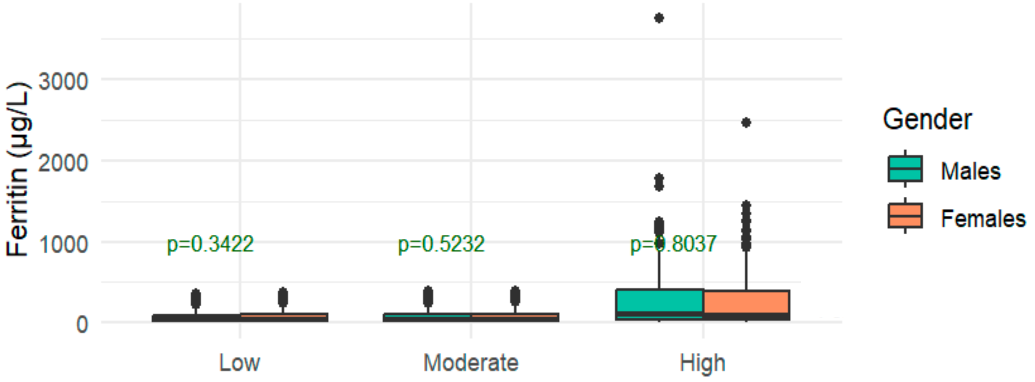

Supplementary Figure S1: Boxplot of Ferritin Levels Across Red Meat Consumption Categories by Gender.

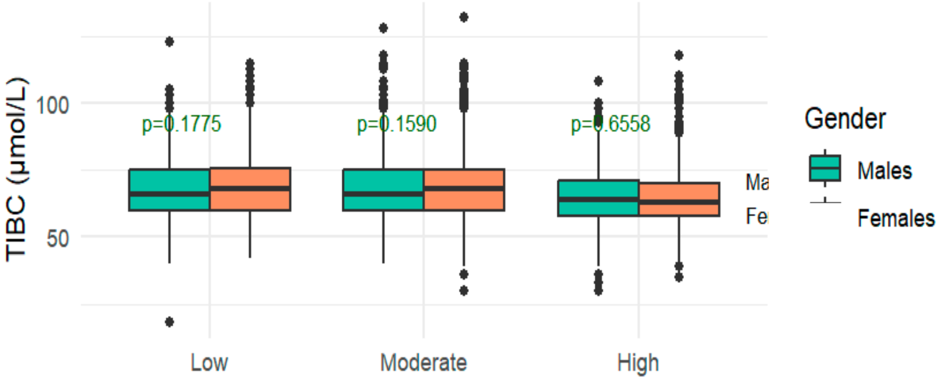

Supplementary Figure S2: Boxplot of TIBC Levels Across Red Meat Consumption Categories by Gender.
